# How Easy Is It to Read Websites About Schizophrenia?

**DOI:** 10.7759/cureus.48790

**Published:** 2023-11-14

**Authors:** Kerem Laçiner, Yiğit Şenol

**Affiliations:** 1 Psychiatry, Kanuni Sultan Süleyman Training and Research Hospital, Istanbul, TUR; 2 Public Health, Afyonkarahisar Provincial Health Directorate, Afyonkarahisar, TUR

**Keywords:** web search, public health informatics, readability, internet, schizophrenia

## Abstract

Unlike legibility, which is determined according to the formal characteristics of a text, such as typeface and page shape, 'readability' defines whether the text is easy to follow and understand by the reader. In addition, readability refers to the level of education necessary for comprehending a given text. The readability of a text can be objectively measured using mathematical formulas based on the relationships between the number of syllables, words and sentences it contains. In order for schizophrenia patients and their relatives to understand their current situation and what kind of process awaits them and to adapt to treatment, the informative texts on websites should be easy to read. Our study aimed to examine the texts about schizophrenia on websites in terms of Turkish readability levels. For the study, 'schizophrenia' was typed into the Google search engine, and the first 50 eligible websites were included. Web pages were analysed in three groups: hospitals, websites created by physicians specializing in psychiatry and other websites. In our study, formulas developed by Ateşman and Bezirci-Yılmaz were used to evaluate readability levels. Statistical analyses used analysis of variance (ANOVA) to compare normally distributed groups of three. There was no statistically significant difference between the readability of the three groups (p>0.05). Our research discovered that the initial 50 websites inspected concerning schizophrenia contained texts that were challenging to read, requiring a minimum of 12 years of education. The readability level of schizophrenia-related websites was observed to be much higher than the average education level in Turkey. This situation may pose difficulties for individuals with schizophrenia and their relatives to get information about schizophrenia online. In addition, if the general population has more accurate information about schizophrenia and can understand it correctly, it may reduce the wrong attitudes and stigmatization towards individuals with schizophrenia.

## Introduction

Schizophrenia is a clinical syndrome with high morbidity and mortality caused by a dynamic interaction of genetic and environmental risk factors. The first hospitalisation of patients with schizophrenia usually occurs in adolescence or early adulthood with positive symptoms, such as hallucinations and/or delusions, disorganised speech or disorganised behaviour. Negative symptoms, characterised by reduced or absent normal behaviour and functioning related to motivation, interest and verbal or emotional expression, are also present in most patients from the first admission. In addition, it has been reported that language use, motor activity, voluntary behaviour, affective symptoms, cognitive symptoms such as attention, memory, executive function, verbal fluency and learning difficulties are present in most people with schizophrenia before the onset of the illness, as are negative symptoms [1-2].

Schizophreniaʼs wide range of symptoms characterises it as a syndrome that is not a single disease but an entity with many clinical manifestations. Its complex nature makes it challenging for the general public to understand and accept, leading to frequent stigmatisation [3]. Individuals diagnosed with schizophrenia and their families are often exposed to prejudiced societal attitudes, especially during the first onset of symptoms. This situation makes it difficult for patients and their relatives to accept the disease and the treatments necessary for recovery [4]. Misinformation or misconceptions about an illness can impact the help-seeking patterns of families and their attitudes and behaviours towards patients [5].

Research on attitudes towards mental illness and stigma emphasises that the primary factor influencing attitude change is the source of information [6]. The Internet is now widely recognised as the primary source of health information [7]. Therefore, the information about schizophrenia on the Internet must be accurate in content, sensitive to prevent stigmatisation and written in plain language.

The average adult in the United States has an eight-grade education, and therefore, the National Institutes of Health (NIH) and the American Medical Association (AMA) recommend that patient materials be readable at ≤sixth-grade reading level [8]. In 2016, Yeşilyurt et al. conducted a study called 'Average and Expected Years of Schooling by Provinces in Turkey' and they reported that individuals aged 25 and above in Turkey averaged 6.51 years of schooling [9].

Unlike legibility, which is determined according to the formal characteristics of a text, such as typeface and page shape, ʻreadabilityʼ defines whether the text is easy to follow and understand by the reader. In addition, readability refers to the level of education necessary for comprehending a given text. The readability of a text can be objectively measured using mathematical formulas based on the relationships between the number of syllables, words and sentences it contains [10].

Numerous studies have evaluated the readability of scientific texts. Some of these specifically focus on assessing the readability of consent forms and informational texts intended for patients, highlighting the importance of providing accurate information [11-12]. Studies have also been conducted on the readability of drug package inserts prepared for patients [13].

Considering that patients with schizophrenia and their relatives will search for information about this disease on the Internet after they are diagnosed, it is of great importance that the information on these sites is easy to read so that patients and their relatives can understand their current situation, the process awaiting them and the treatment. 

In addition, considering the inadequacies in the cognitive skills of individuals with schizophrenia, the importance of writing informative texts about this disease in an easy-to-understand manner increases even more. Our study examined the texts of the first 50 websites that appeared after typing ʻschizophreniaʼ into the Google search engine in terms of Turkish readability levels. Thus, it will be possible to evaluate which websites frequently visited by schizophrenia patients and their relatives to get information about this disease contain text appropriate for various educational levels.

## Materials and methods

Readability metrics

When evaluating written text for readability, various mathematical formulas are used that are based on the relationships among the number of syllables, words and sentences contained within the text. A text that contains long sentences with frequent use of polysyllabic words will result in low readability. Many globally recognised readability metrics have been developed for this purpose [14]. The main formulas utilised in this study for evaluating Turkish text readability levels were the indices produced by Ateşman and Bezirci-Yılmaz, whose scientific efficiency has been established [10-15].

In Ateşman’s readability formula, the calculation produces a score between 0 and 100. The higher the score, the more straightforward the writing. Ateşman additionally published Table 1, which indicates the education level for which the text is readable based on the formula’s resulting score.

**Table 1:** The score measured in the Ateşman readability formula is the level of readability and the corresponding education level Source: [15]

Points	Readability level	Education level
90–100	Very easy	Readable by an individual with a primary school education of 4th grade and below
80–89	Easy	Readable by an individual with a 5th or 6th grade education
70–79		Readable by an individual with a 7th or 8th grade education
60–69	Medium difficulty	Readable by an individual with a 9th or 10th grade education
50–59		Readable by an individual with an 11th or 12th grade education
40–49	Difficult	Readable by an individual with a 13th to 15th grade education
30–39		Readable by an individual with an undergraduate education
≤29	Very difficult	Readable by an individual with a postgraduate education

Ateşman’s readability formula is as follows: readability score = 198.825 - 40.175 x word length (total syllables / total words) - 2.610 x sentence length (total words / total sentences) [15].

Bezirci-Yılmaz developed a formula to determine a text’s readability level by considering the number of words and syllables in a sentence [10]. The result obtained from this formula indicates the educational level for which the text is readable (see Table 2).

**Table 2:** Education level corresponding to the points/grade obtained through the Bezirci-Yılmaz readability formula Source: [10]

Grade	Education level
1–8	Primary school
9–12	Middle school (High school)
12–16	Further education
16+	Academic-level education

Bezirci-Yılmaz’s readability formula is as follows: readability score = √OKS x [(H3 x 0.84) + (H4x1.5) + (H5x3.5) + (H6x26.25)], where *OKS* is the average word count, *H3* is the average number of words with three syllables, *H4* is the average number of words with four syllables, *H5* is the average number of words with five syllables and *H6* is the average number of words with six or more syllables.

Selection of websites

For this study, we typed 'schizophrenia' in Turkish in the Google search engine between Septermber 1, 2023 and September 15, 2023. The first 50 website results were included in the study after excluding websites that appeared more than once because they were shared in the search engine with advertising sponsorship.

The researchers recorded the sites that appeared as a result of the first search, a list was prepared and we examined the list as researchers. The web pages were divided into three groups and then analysed. The three groups were 1) institutional structures such as hospitals and similar healthcare organisations; 2) websites created by physicians specialising in psychiatry; and 3) web pages that contain general informative content, such as newspaper articles and blog posts.

In making this categorisation, we made the following distinction: we assumed that psychiatrists are mental health professionals and can make a difference when preparing such texts compared to non-mental health professionals. In addition to mental health professionals, there may be professionals in the group of hospitals who can make the necessary corrections and edits to prepare a text. The grouping was based on general search results.

Statistical analysis

Descriptive statistics, including percentages, frequencies, means, medians, maximums and minimums, were determined from the study. The Shapiro-Wilk Test was used to evaluate the normal distribution properties of continuous data, which were found to show a normal distribution. To compare the three groups with a normal distribution in statistical analyses, analysis of variance (ANOVA) was implemented, with p<0.05 set as the statistical significance threshold.

## Results

We assessed 50 websites for their readability. Of these, 38% (n = 19) were hospital sites, 24% (n=12) were physician sites and 38% (n = 19) were news and blog posts. Table 3 compares the readability of hospital, physician and other websites according to the Bezirci-Yılmaz and Ateşman formulas. No statistically significant differences were found between the groups (p>0.05).

**Table 3:** Comparison of the readability of hospital, physician and other websites The data have been represented as N, %, mean ± SD (standard deviation). p<0.05 was set as the statistical significance threshold.

N % Mean SD p					
Bezirci-Yılmaz					
Hospital	19	38	12.59	3.15	0.625
Physician	12	24	12.34	2.17	
Other	19	38	11.82	1.82	
Total	50	100	12.24	2.46	
Ateşman					
Hospital	19	38	44.84	8.85	0.637
Physician	12	24	45.50	6.39	
Other	19	38	47.00	5.33	
Total	50	100	45.82	7.02	

According to the Bezirci-Yılmaz readability formula, the highest readability rate for the evaluated websites was at the high school (46%) and undergraduate (42%) levels (refer to Table 4).

**Table 4:** Readability level of websites according to the Bezirci-Yılmaz formula The data have been represented as N and %.

Readability level	n	%
Primary school	2	4.0
Middle school (high school)	23	46.0
Further education	21	42.0
Academic-level education	4	8.0

According to the Ateşman readability formula evaluation of website readability levels, most of the text analysed was difficult (70%). There was no text categorised as easy or very easy (as shown in Table 5).

**Table 5:** Readability level of websites according to the Ateşman formula The data have been represented as N and %.

Readability level	n	%
Very difficult	1	2.0
Difficult	35	70.0
Medium difficulty	14	28.0

## Discussion

The primary objective of schizophrenia treatment is the rapid elimination of psychotic symptoms without undue impact on the patient. Antipsychotic treatment is crucial for symptom control and achieving remission. To accomplish these goals, conventional treatments, including antipsychotic medication, psychological interventions and social support, are utilised. As clinical knowledge and practices surrounding schizophrenia have advanced, clinicians and mental health centres focusing on schizophrenia recovery seek to achieve more than symptom reduction. They promote functionality, well-being, coping skills and quality social relationships. Psychotherapy and psychosocial rehabilitation are vital components in achieving these goals. Psychoeducation is a fundamental component of psychotherapeutic interventions and programs for psychosocial rehabilitation [16].

Today, psychoeducation is commonly used as a treatment approach for individuals with schizophrenia and their families, with a focus on providing systematic education about the aetiology and diagnosis of the illness, prognosis, available treatment options, potential medication side effects, challenges that may arise during the treatment process and coping strategies and patients’ rights [17]. Although conveying accurate information through the Internet cannot replace psychoeducation as a treatment method, it has the potential to contribute to all targeted elements and reduce social stigmatisation from caused by misinformation about the disease.

There is limited research available on the readability of Turkish websites. In 2020, Çifci et al. [18] conducted a study evaluating the readability of Turkish websites related to substance addiction using the Ateşman and Bezirci-Yılmaz readability formulas. The results indicated that these websites are readable with an average of 14 years of education.

In Solak et al.’s [19] study in 2021 evaluating smoking cessation websites, the Bezirci-Yılmaz readability index revealed that these websites required approximately 13 years of education to be readable. Likewise, in a related study by Solak et al. in 2019, examining websites about colorectal cancer, the Bezirci-Yılmaz index deemed readability achievable with approximately 12 years of education [20]. In a study by Tahir et al. in 2021 [21], the Ateşman and Çetinkaya readability formulas were used to assess the readability level of websites related to dizziness. The texts were found to be readable with an average of eight to nine years of education.

In the global literature about the readability of schizophrenia-related online content, Kalk et al. [22] conducted a study in 2008. According to the Standardized Flesch Reading Ease classification, the study reported that 40% of the evaluated sites were very difficult, 55% were difficult and 5% were fairly difficult. None of the selected sites were deemed easy to comprehend. The findings of the study were based on the results of the PRODIGY study, a comprehensive report on the readability, comprehensibility and effectiveness of patient information leaflets in the United Kingdom (UK), which completed Phase 1 studies between 1995 and 1998 and Phase 2 studies in 1999, and assumed an average reading age of 12 years in the UK [22-23]​​​​​​​​​​​​​​​​​​​​​.

In the literature, there are also studies on videos as a channel for obtaining information about schizophrenia on the Internet. In 2022, Kahve et al. [24] examined the quantitative and qualitative characteristics of YouTube videos about schizophrenia, assessing the quality of the information provided, which videos were watched the most and whether there was a relationship between the popularity and quality of the videos. The researchers noted that the quality of the videos was low and concluded that this could lead to inaccurate attitudes about the disease and its treatment. They also emphasised that misinformation about the disease may contribute to the stigma surrounding the disease. They stated that there is a need for mental health professionals to be more visible in YouTube videos and to provide quality information in the videos [24]​​​​​​​.

In 2017, Kugar et al. [23] conducted an Internet search to gauge the readability of educational texts for mental health patients from top psychiatric hospitals and the Veterans Health Administration in the United States. The authors of that study discovered that the average readability score for mental health information on all websites examined was 9.52. The average American adult’s educational level is at a seventh-grade reading level, and therefore, the NIH and AMA recommend that informational texts should be written at a sixth-grade reading level. This study emphasised that online resources regarding mental health disorders exceed the NIH and AMA’s recommended level of complexity [25].

Our research discovered that the 50 websites inspected concerning schizophrenia contained texts that were challenging to read, requiring a minimum of 12 years of education. Most are written at a level equivalent to that of a high school graduate, with only a few informative texts aimed at lower education levels. Given that the average number of years of education in Turkey is 6.5, it is apparent that these texts’ are exceedingly difficult for the average population to read [9]. No significant difference was found in website readability among the three groups analysed in our study: hospitals, physicians and other entities. Given their facilities, institutions like hospitals should be more attentive to this aspect.

In addition, health system administrators must expand psychoeducational interventions for patients and their relatives as a public health policy and for mental health professionals to be more visible in all kinds of media where they can provide information about the disease. Such interventions can reduce misinformation about schizophrenia and related stigmatisation and increase the literacy of the whole community, especially patients and their relatives, regardless of their years of education, about schizophrenia on the Internet.

Limitations

Our study has limitations. We evaluated only the first 50 websites; adding additional sites could have made the research more inclusive. In a search engine, relevant websites may vary based on users' past searches. We have not used a specific browser to standardize this. In addition, the readability formulas used in our study focus on quantitative aspects, such as word count, syllables and sentence structure, rather than qualitative features related to the language. Thus, our study results do not comprehensively assess textʼ readability. For this purpose, a more comprehensive evaluation could have been conducted by qualitatively examining the relevant texts and having them read by patient populations.

## Conclusions

Our study indicates that the readability level of schizophrenia-related websites is above the average educational level in Turkey, which creates difficulties in online access for individuals with schizophrenia and their families regarding comprehending relevant information. Increased accuracy and comprehension of schizophrenia-related information among the general public may reduce prejudice and negative attitudes towards those affected by the disorder.

## References

[1] Patel KR, Cherian J, Gohil K, Atkinson D (2014). Schizophrenia: overview and treatment options. P T.

[2] Owen MJ, Sawa A, Mortensen PB (2016). Schizophrenia. Lancet.

[3] Vrbova K, Prasko J, Holubova M (2016). Self-stigma and schizophrenia: a cross-sectional study. Neuropsychiatr Dis Treat.

[4] Buizza C, Schulze B, Bertocchi E, Rossi G, Ghilardi A, Pioli R (2007). The stigma of schizophrenia from patients' and relatives' view: A pilot study in an Italian rehabilitation residential care unit. Clin Pract Epidemiol Ment Health.

[5] Yıldız M, Yazıcı A, Çetinkaya Ö, Bilici R, Elçim R (2010). Relatives' knowledge and opinions about schizophrenia [Article in Turkish]. Türk Psikiyatri Dergisi.

[6] Li J, Zhang MM, Zhao L, Li WQ, Mu JL, Zhang ZH (2018). Evaluation of attitudes and knowledge toward mental disorders in a sample of the Chinese population using a web-based approach. BMC Psychiatry.

[7] Litzkendorf S, Frank M, Babac A, Rosenfeldt D, Schauer F, Hartz T, Graf von der Schulenburg JM (2020). Use and importance of different information sources among patients with rare diseases and their relatives over time: a qualitative study. BMC Public Health.

[8] Eltorai AE, Naqvi SS, Ghanian S, Eberson CP, Weiss AP, Born CT, Daniels AH (2015). Readability of invasive procedure consent forms. Clin Transl Sci.

[9] Yeşilyurt ME, Karadeniz O, Gülel FE, Çağlar A, Uyar SG (2016). Mean and expected years of schooling for provinces in Turkey [Article in Turkish]. Pamukkale Journal of Eurasian Socioeconomic Studies.

[10] Bezirci B, Yilmaz AE (2010). A software library for measurement of readability of texts and a new readability metric for Turkish [Article in Turkish]. Dokuz Eylül Üniversitesi Mühendislik Fakültesi Fen ve Mühendislik Dergisi.

[11] Dağdelen C, Erdemoğlu E (2023). Determination of the readability level of consent forms used in the gynecology and obstetrics clinic at Suleyman Demirel University. Cureus.

[12] Dural İE (2022). Are consent forms used in cardiology clinics easy to read?. Turk Kardiyol Dern Ars.

[13] Ay İE, Duranoğlu Y (2022). An evaluation of the readability of package inserts of eye drops [Article in Turkish]. Anatolian Clinic the Journal of Medical Sciences.

[14] FL R (1948). A new readability yardstick. J Appl Psychol.

[15] Ateşman E (1997). Measurement of readability in Turkish [Article in Turkish]. Dil Dergisi.

[16] Chien WT, Leung SF, Yeung FK, Wong WK (2013). Current approaches to treatments for schizophrenia spectrum disorders, part II: psychosocial interventions and patient-focused perspectives in psychiatric care. Neuropsychiatr Dis Treat.

[17] Rummel-Kluge C, Kissling W (2008). Psychoeducation in schizophrenia: new developments and approaches in the field. Curr Opin Psychiatry.

[18] Çifci HK, Kozanhan B, Solak İ (2020). Evaluation of readability of Turkish websites on substance addiction [Article in Turkish]. Bağımlılık Dergisi.

[19] Solak İ, Kozanhan B, Poçanoğlu AA, Gürer N, Enes A, Eryılmaz M (2021). Evaluation of readability of Turkish websites about smoking cessation. Bağımlılık Dergisi.

[20] Solak M (2019). Readability of websites containing information about colorectal cancer [Article in Turkish]. Harran Üniversitesi Tıp Fakültesi Dergisi.

[21] Tahir E, Kent AE (2021). Readability analysis of internet-based patient information regarding dizziness [Article in Turkish]. KBB-Forum.

[22] Kalk NJ, Pothier DD (2008). Patient information on schizophrenia on the internet. Psychol Bull.

[23] Wilson R, Kenny T, Clark J, Moseley D, Newton L, Newton D, Purves I (1998). PIL's Project summary report: ensuring the readability and understandability and efficacy of patient information leaflets. SCHIN.

[24] Kahve AC, Gonca A, Hasan K, Hacımusalar Y (2022). What does YouTube® say about schizophrenia: is it a reliable source of information?. Acıbadem Üniversitesi Sağlık Bilimleri Dergisi.

[25] Kugar MA, Cohen AC, Wooden W, Tholpady SS, Chu MW (2017). The readability of psychosocial wellness patient resources: improving surgical outcomes. J Surg Res.

